# An Integrated Prehospital Point‐of‐Care Lung Ultrasound Protocol for Patients With Dyspnea

**DOI:** 10.1002/jum.70218

**Published:** 2026-03-07

**Authors:** David Purkarthofer, Marco Seebacher, Michael Furtmüller, Jakob Laumer, Peter Zechner, Otmar Schindler, Geza Gemes, Michael Eichlseder

**Affiliations:** ^1^ Medical University of Graz Graz Austria; ^2^ Medizinercorps Graz Austrian Red Cross Graz Austria; ^3^ Department for Health Studies FH JOANNEUM University of Applied Sciences Graz Austria; ^4^ University Center for Acute Care Medicine LKH University Hospital Graz Graz Austria; ^5^ Division of Anaesthesiology and Intensive Care Medicine 1, Department of Anaesthesiology and Intensive Care Medicine Medical University of Graz Graz Austria; ^6^ Department of Cardiology and Intensive Care LKH Graz II Graz Austria; ^7^ Department of Internal Medicine and Pulmonology LKH Hochsteiermark Leoben Austria; ^8^ Department of Anaesthesiology and Intensive Care Medicine LKH Hochsteiermark Leoben Austria

**Keywords:** dyspnea, emergency medicine, lung ultrasound, paramedic, point‐of‐care ultrasound, prehospital care

## Abstract

We present a time‐efficient, clinically integrated lung ultrasound protocol tailored for prehospital use by providers not yet routinely using ultrasound. Specifically designed to support the recognition of pneumothorax and interstitial syndrome, the protocol emphasizes operational feasibility, spectrum‐based diagnostic reasoning, and strict time limitations to avoid delays in definitive care. It includes standardized scanning sequences, documentation, and a governance framework for training, certification, and quality assurance. Developed and implemented in an Austrian ambulance service, it may serve as a scalable model for expanding ultrasound access. A prospective registry supports the evaluation of feasibility, image quality, diagnostic yield, and clinical impact.

**Abbreviation**
POCUS: point‐of‐care ultrasound

Current expert consensus affirms that lung ultrasound is both feasible and valuable in the prehospital setting, but emphasizes the need for structured frameworks to guide safe integration and future development.^1^ In response, we present a comprehensive, operationally integrated lung ultrasound protocol designed for prehospital providers, who are not yet routinely using lung ultrasound, and which was implemented at an Austrian ambulance service. This protocol goes beyond a simple scanning algorithm—it introduces a multi‐domain innovation that includes: technical guidelines optimized for out‐of‐hospital conditions, a time‐capped algorithm with predefined stopping criteria, clinical decision‐making support based on a spectrum‐based reasoning model, and a governance and recertification framework that ensures safe, sustainable implementation by providers not using lung ultrasound regularly in their current clinical practice. This group may include most paramedics, but also other pre‐ and in‐hospital care providers, including nurses or junior physicians.

Point‐of‐care ultrasound (POCUS) is increasingly recognized as a valuable diagnostic tool in prehospital emergency medicine. Advances in portable ultrasound technology and a growing body of clinical evidence have enabled its integration into care environments that were previously considered impractical for imaging.^2^, ^3^

Despite this progress, the use of diagnostic ultrasound in prehospital care remains largely restricted to physicians in many healthcare systems. However, a number of paramedic‐led services have successfully implemented POCUS protocols, demonstrating the feasibility of expanding ultrasound access through targeted training and standardized procedures.^4^, ^5^, ^6^, ^7^, ^8^

Among the various applications of prehospital ultrasound, lung imaging in patients with dyspnea is particularly promising. For decades, the lung was considered a blind spot for ultrasound due to the air–tissue interface. This perception changed with the early work demonstrating the potential in detecting various pathologies^9^ and the introduction of the BLUE protocol in 2008,^10^ which demonstrated the feasibility of structured lung ultrasound in acute care. Since then, multiple studies—including 2 international consensus papers by ultrasound experts—have confirmed the diagnostic speed and accuracy of focused lung ultrasound in emergency settings.^1^, ^11^, ^12^, ^13^, ^14^, ^15^

Importantly, focused lung ultrasound can be performed effectively after relatively limited training,^12^ supporting its potential role for providers not using it in their current clinical practice.

Most evidence to date originates from emergency departments, where lung ultrasound has been shown to be at least as accurate as diagnostic workups involving chest x‐rays and natriuretic peptides for identifying interstitial syndrome due to acute heart failure.^11^, ^12^, ^16^, ^17^ Diagnostic performance also shows a strong correlation with computed tomography findings.^18^ As these imaging and testing modalities are not available in the prehospital setting, POCUS may offer significant added value in differentiating respiratory pathologies early in the care pathway. Data from a small prehospital study and several case reports suggest that ultrasound can support differentiation between acute heart failure and exacerbations of chronic obstructive pulmonary disease.^8^, ^19^

At the same time, delays in transport continue to pose a critical risk in prehospital emergency care. While no specific outcome data are available for the broader cohort of patients presenting with dyspnea, Żurowska‐Wolak et al demonstrated that in patients with ST‐segment elevation myocardial infarction—a condition that may also present with dyspnea—prolonged prehospital time was independently associated with increased in‐hospital mortality and reduced left ventricular ejection fraction at discharge. Notably, each additional minute of delay translated to approximately a 2% higher risk of adverse outcomes.^20^ Although these findings cannot be directly extrapolated to all patients with dyspnea, they underscore the critical importance of minimizing time to definitive care. In the absence of more specific data, and based on clinical reasoning and the availability of superior in‐hospital diagnostics and interventions, timing should be considered a priority in this patient group as well. Accordingly, any additional diagnostic intervention, such as prehospital ultrasound, must be carefully evaluated for its potential to delay transport and definitive treatment.

## Key Innovations

This protocol goes beyond a simple scanning algorithm. It introduces a multi‐domain innovation that includes: technical guidelines optimized for out‐of‐hospital conditions, a strict 3‐minute time‐capped algorithm with predefined early termination criteria, clinical decision‐making support based on a spectrum‐based reasoning model, and a governance and recertification framework enabling safe, sustainable use by providers without routine lung ultrasound practice. To our knowledge, no prior prehospital lung ultrasound protocol combines these elements into a single, operationally integrated package. This approach directly addresses 2 persistent barriers to wider prehospital ultrasound adoption: the risk of delaying definitive care and the lack of structured support for novice operators.

## Methods

The protocol was developed through a structured, multi‐step process to ensure clinical relevance, operational feasibility in the prehospital setting, and practical applicability for trained novice providers. It was primarily developed for the Medizinercorps Graz of the Austrian Red Cross,^21^ an advanced care ambulance service operating in a medium‐sized European city. Team leaders of the 4‐person ambulance crews hold the highest non‐physician qualification in Austria, with a minimum training duration of more than 3000 hours. Their scope of practice includes administering an extensive predefined list of medications, performing prehospital point‐of‐care blood testing, and providing non‐invasive and invasive ventilation, among other interventions. The protocol is designed to be transferable to a wide range of systems staffed by providers without previous routine lung ultrasound practice. The protocol was implemented on September 1, 2025 and has been in use since then.

### Evidence Base and Protocol Design

The protocol was informed by a scoping review conducted by Ovesen et al,^22^ which categorized the existing literature on focused lung ultrasound according to diagnostic efficacy and clinical impact, using the Fryback and Thornbury model.^23^ This structured evidence map enabled the targeted selection of studies on lung ultrasound in patients with acute dyspnea, with a particular focus on those reporting effects on clinical decision‐making and patient outcomes. The interactive format of the database allowed for efficient identification of relevant studies, excluding those combining multiple ultrasound modalities, and prioritizing those with high diagnostic yield, therapeutic relevance, or larger sample sizes (≥100 patients).

Building on this evidence base, we developed a pragmatic ultrasound protocol aimed at detecting key sonographic signs of prehospitally treatable, time‐critical conditions in dyspneic patients, primarily pneumothorax and interstitial syndrome secondary to acute heart failure. Each component of the scanning sequence and interpretative logic was evaluated for its diagnostic yield, anticipated scan duration, and usability by providers with limited ultrasound experience.

### Adaptation to Operational Requirements and Constraints

The protocol was specifically adapted to prehospital operational constraints, including time pressure, variable patient positioning, suboptimal lighting, and limited access to the patient during transport. As a result, scanning approaches requiring access to posterior lung zones were omitted to ensure feasibility under real‐world conditions.

To further mitigate these challenges, a mandatory pre‐scan assessment ensures that ultrasound is used only when it is likely to provide diagnostic benefit without delaying critical interventions or transport. The scan is explicitly time‐limited to 3 minutes and incorporates predefined stopping criteria if no abnormalities are detected in the initial views.

### Training Considerations

The protocol is designed for use by prehospital care providers with basic to intermediate ultrasound training. Anatomical landmarks and sonographic signs were selected to promote consistent image acquisition and minimize the risk of misinterpretation. A longitudinal probe orientation was chosen, as it reliably facilitates pleural line identification between rib shadows—both in field conditions and during retrospective expert review.^24^

## Ultrasound Protocol

**Figure 1:**
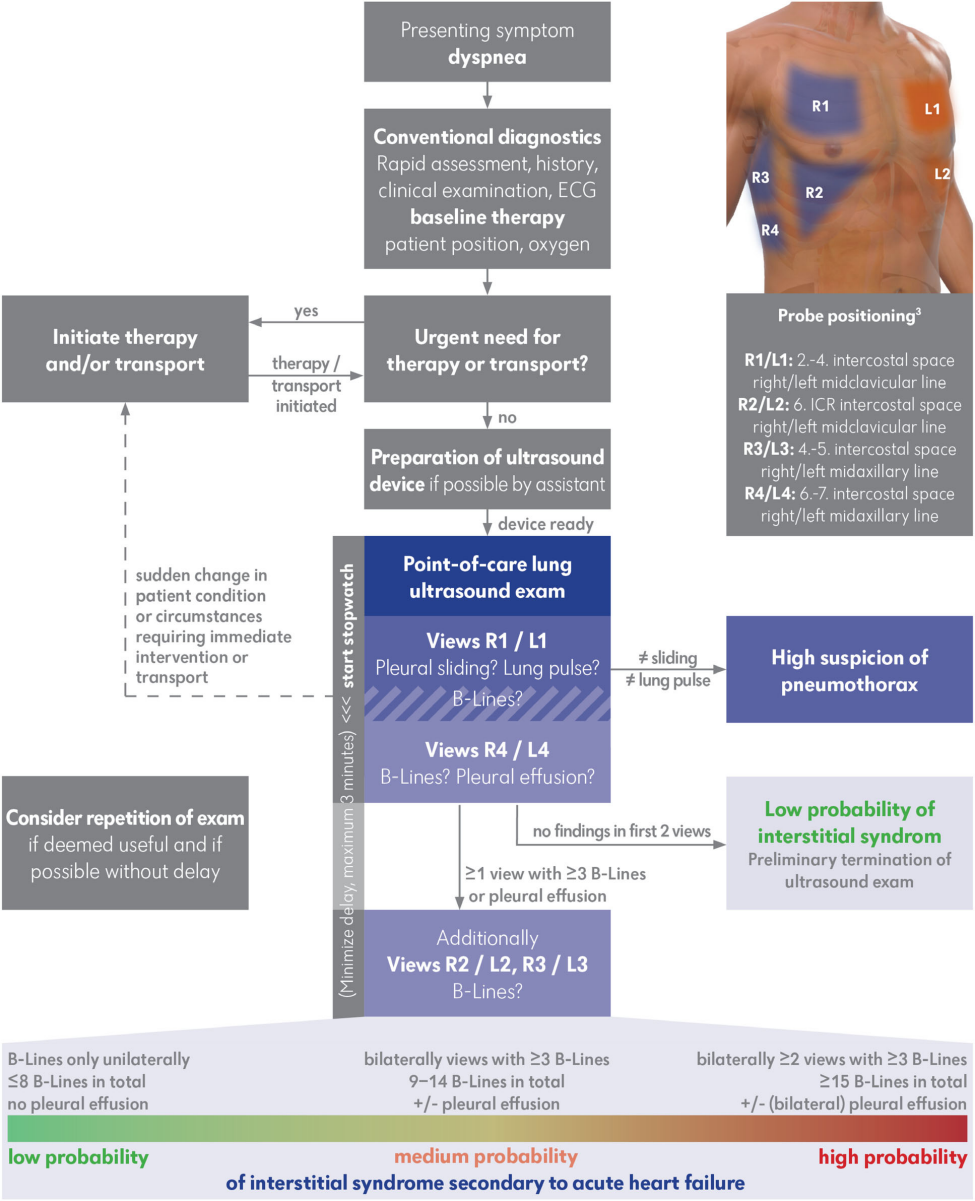
Algorithm for pre‐hospital point of care ultrasound for patients with dyspnea.

The protocol is structured as a focused lung ultrasound examination, with a limit of adding no more than a maximum of 3 minutes to on‐scene and transport time. It is intended for use during phases of care where it can be utilized with no or minimal time delays—such as while awaiting transport readiness or, if appropriate, during transport. The scanning sequence follows a fixed anatomical order and uses a longitudinal (sagittal) probe orientation with the marker directed cranially to support consistent acquisition and reproducible interpretation (see Figure 1).

### Initial Safety and Timing Assessment

Prior to initiating the scan, providers must assess whether any ongoing diagnostic or therapeutic interventions, particularly patient transport, take precedence. Lung ultrasound is intended for use when potentially treatable causes of dyspnea, such as interstitial syndrome secondary to acute heart failure or pneumothorax, are considered likely differential diagnoses.

### Scanning Sequence and Anatomical Zones

**Figure 2:**
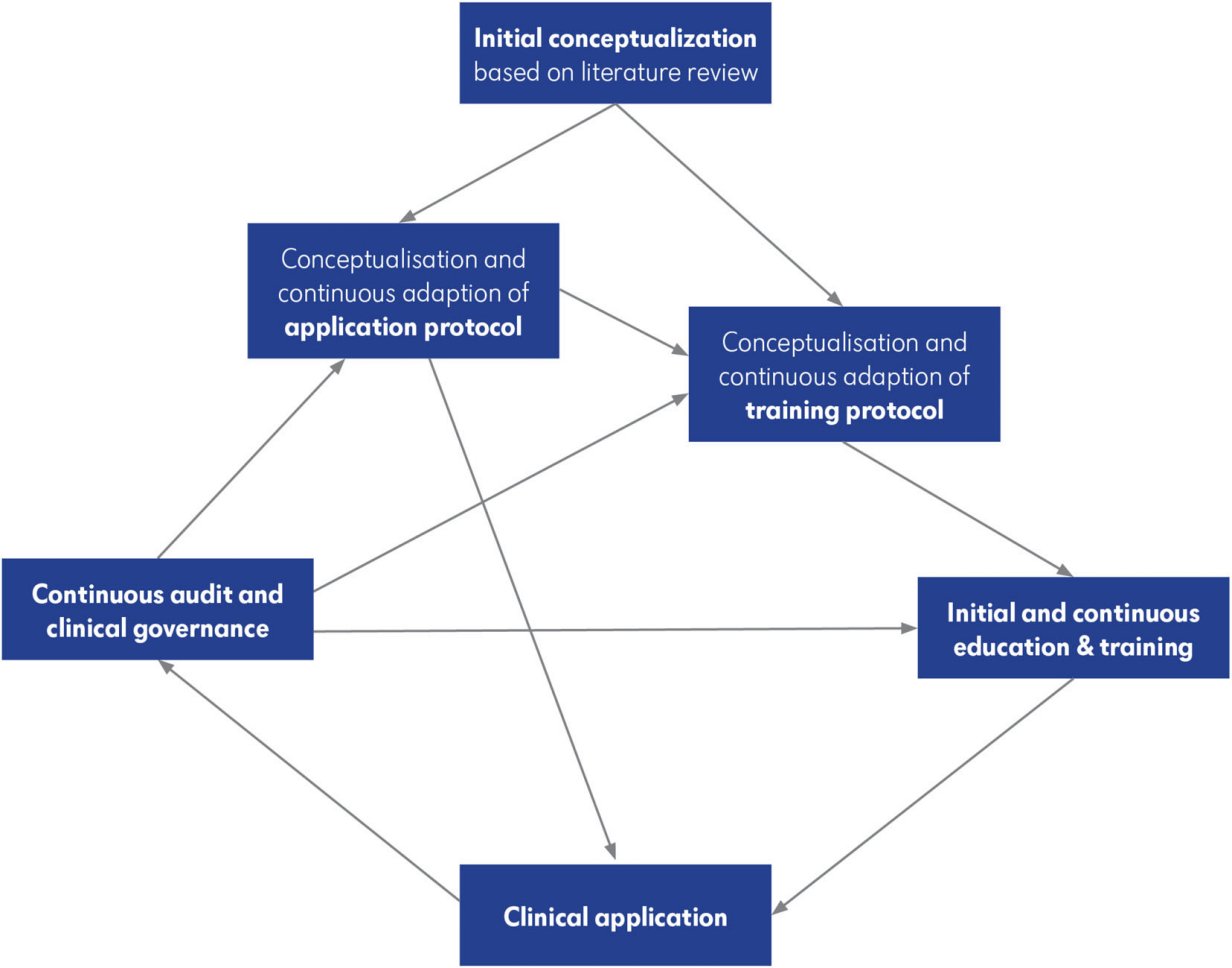
Flowchart of conceptual framework for protocol adaptation. Case reviews inform ongoing training and iterative protocol updates.

The scan begins with bilateral assessment of the anterior upper chest—typically the second to fourth intercostal spaces along the midclavicular line (zones R1/L1; see Figure 2). This region offers high sensitivity for detecting pneumothorax. Pleural sliding is assessed over at least 1 complete respiratory cycle.

This view also serves as the initial assessment zone for interstitial syndrome. Providers document the presence and number of B‐lines observed in each region.

The next view targets the lateral lower thorax—typically the sixth intercostal space in the midaxillary line (zones R4/L4) to evaluate for B‐lines and pleural effusions. The probe is moved caudally until the liver or spleen is visualized to assess for fluid in the costophrenic angle.

If no abnormalities, including B‐lines, are observed in these 2 bilateral zones, the scan may be concluded early. In such cases, continued scanning is unlikely to reveal findings suggestive of interstitial syndrome, and transport or other medical interventions should proceed without delay.

If abnormalities are detected, additional views are obtained from the anterior lower thorax (approximately sixth intercostal space, midclavicular line; zones R2/L2) and the lateral upper thorax (approximately fourth intercostal space, midaxillary line; zones R3/L3).

In each zone, providers document the number of B‐lines, as well as the presence or absence of pleural sliding and pleural effusion. Additional findings—such as localized consolidations suggestive of pneumonia or pulmonary infarction—may be noted if observed but should not be actively sought outside the defined protocol zones, in order to avoid unnecessary delays.

### Documentation and Interpretation

All findings are documented using a structured format (Table 1), including the following core elements:

- Pleural sliding: present or absent (right and left).
- if absent: Lung point present or absent.
- B‐lines: number per region, total count, and number of “positive” zones (≥3 B‐lines per zone).
- Pleural effusion: present or absent (right and left).

**Table 1:** Data Collected for Each Lung Ultrasound Application

Field	Values	Note
Basic demographic data		
Patient sex	Male/female	
Patient age	Integer	
Dispatch and attendance of units	ALS ambulance/ALS ambulance + physician response unit/Primarily ALS ambulance, physician response unit activated from scene	
Reason/code for dispatch	DIASweb® standardized calltaker coding (free text if not available)	
Transport to hospital	Yes/no	
Suspected diagnosis	Choice of diagnosis from 100+ options in 13 categories; free text if no applicable option	
NACA score	Choice from 0 to 7	0: patient without injury/disease
Diagnostics and interventions		
12‐lead ECG performed	Yes/no	
i.v. access obtained	Yes/no	
i.o. access obtained	Yes/no	
Non‐invasive ventilation	Yes/no	
Endotracheal intubation	Yes/no	
POC blood testing	Yes/no	
Pharmaceutical therapy	None/Within paramedic scope/outside paramedic scope, by physician	
RSI (with physician attending)	Yes/no	
POCUS exam data		
POCUS location	On scene/during transport	Multiple choice possible
Provider perceived impact on differential diagnosis	Confirmed suspected diagnosis/excluded considered diagnosis/unexpected diagnosis/no impact	Multiple choice possible
Provider perceived impact on treatment strategy	Reinforcement for planned treatment strategy/change of the planned treatment strategy/no impact	
POCUS duration (seconds)	Integer	Or measurement impossible
POCUS protocol completed	Yes/no	
Reason why protocol was not completed	Free text	Active when previous no
Lung exam (for each field: R1‐R4, L1‐L4)		
Field examined	Yes/no	
Pleural sliding	Yes/no	Active when examined
B‐Lines	Integer	Active when examined
Lung point	Yes/no	Active when examined
Ultrasound findings		
No findings	Yes/no	
Pneumothorax	Yes/no	
Interstitial syndrome	Yes/no	
Pleural effusion	Yes/no	
Subpleural consolidation	Yes/no	
Other findings	Yes/no	
Other findings: Specification	Free text	Active when previous yes
Ultrasound re‐examination performed?	Yes/no	
Result of re‐examination	Same as previous/changes	Active when previous yes
Changes from first examination	Free text	Active when previous changes
Comments	Free text	Non‐mandatory field

All examinations are also recorded and stored as ultrasound video loops.

Sonographic findings must always be interpreted in conjunction with the clinical presentation and any additional available data. Absence of pleural sliding is suggestive of pneumothorax. A bilateral distribution of multiple zones with 3 or more B‐lines supports a diagnosis of interstitial syndrome, most often due to acute heart failure. Pleural effusions may support this diagnosis but are neither sensitive nor specific in isolation.^10^, ^11^, ^14^, ^25^, ^26^

In contrast to pneumothorax, where a binary yes/no approach is often feasible, and clinical ambiguity typically centers on the need for (pre‐hospital) invasive intervention versus conservative management, the development of interstitial syndrome represents a more gradual and variable process. Its diagnosis often lies within a gray zone, with multiple diagnostic criteria proposed in the literature.^27^, ^28^ This protocol is specifically designed to support spectrum‐based reasoning, enabling providers to assess the likelihood of interstitial syndrome along a clinical continuum rather than relying on binary rules. This reasoning process facilitates the integration of sonographic findings with other clinical data to inform treatment decisions, including pharmaceutical therapy targeting blood pressure optimization and the initiation of non‐invasive ventilation.

## Governance, Continuous Audit, and Scientific Evaluation

The implementation of any new diagnostic protocol in prehospital emergency care necessitates robust clinical governance to ensure patient safety and sustainable integration into routine operations. This lung ultrasound protocol is embedded within a comprehensive framework that addresses training standards, ongoing competence assessment, structured data monitoring, and scientific evaluation. See Figure 2 for an overview on conceptualization and continuous adaptation based on acquired data.

### Training Oversight and Procedural Authorization

All providers complete a structured ultrasound training program consisting of theoretical instruction (approx. 9 hours including 3 hours of flipped‐classroom e‐lectures), supervised hands‐on practice (approx. 11 hours), independent scanning in healthy volunteers (24 exams across different organ systems), and final competency evaluation. Independent use of the protocol is authorized only after successful completion of a standardized assessment covering image acquisition, interpretation, device handling and adherence to procedural time constraints. Certification is valid for 24 months and may be renewed upon participation in at least 3 feedback sessions with experienced physicians which are offered quarterly and are based on anonymized case discussions including sonographic findings.

Standardized documentation templates and an interpretation algorithm are used to promote consistency, minimize subjectivity, and support decision‐making under operational time constraints.

### Case Documentation and Image Archiving

All examinations performed under the protocol are documented electronically, capturing key clinical parameters, scan duration, sonographic findings, and downstream decisions such as therapy initiation or expedited transport. Ultrasound images and video clips are uploaded to a secure cloud platform compliant with applicable data protection laws for subsequent quality assurance and educational review.

### Clinical Governance and Audit

All cases are subject to routine retrospective review by physicians experienced in lung ultrasound. These evaluations serve both as provider feedback and as part of an ongoing audit process. Selected cases are presented and discussed during quarterly feedback sessions with all active protocol users. Data from all cases, including important safety parameters like the time used for the exam as well as diagnostic accuracy parameters, are used to yearly reevaluate and adapt training and application protocols.

### Scientific Evaluation

A prospective observational registry was established to evaluate the protocol's feasibility, completeness, diagnostic yield, and clinical impact. Outcome measures include scan duration, image quality, influence on prehospital decision‐making, the rate of early termination after initial views, and the frequency of abnormal findings. Although significant legal and operational barriers exist, efforts are currently underway to enable correlation with in‐hospital data, including final diagnoses and imaging results.

## Discussion and Future Directions

### Limitations

This manuscript primarily presents a protocol and implementation concept that was developed and deployed within a single prehospital system. While the framework is intended to offer guidance and inspiration for similar services seeking to integrate point‐of‐care ultrasound, its generalizability remains uncertain. Although detailed data on feasibility, safety, and diagnostic performance are being prospectively collected, the protocol has not yet been clinically validated, and no conclusions can be drawn regarding its effectiveness or impact on patient outcomes. Further multi‐center evaluation will be required to determine its broader applicability and clinical utility.

### Potential Benefits

This protocol offers a practical, time‐efficient framework for prehospital lung ultrasound in patients with dyspnea, specifically designed for use by providers without routine lung ultrasound practice. By integrating a strict 3‐minute time limit, early termination logic, spectrum‐based diagnostic reasoning, and a formal governance and recertification process, it represents a novel, operationally feasible model for safe ultrasound expansion. Its structured and focused design is intended to support timely and clinically relevant decision‐making without causing significant delays to definitive care.

In operational terms, this innovation aims to improve diagnostic confidence in the field, guide earlier initiation of targeted therapies, and support more appropriate transport decisions—benefits that may translate into better patient outcomes, even if definitive in‐hospital diagnostics remain superior.

### Future Directions

This approach may serve as a scalable model for other emergency care systems seeking to expand ultrasound capabilities beyond physician‐led services. Integration into structured training curricula—combined with standardized documentation and continuous quality assurance—could facilitate safe and consistent implementation across diverse prehospital settings.

Future research should evaluate the protocol's clinical and operational impact, including effects on decision‐making, resource utilization, and patient outcomes.

## Data Availability

Data sharing not applicable to this article as no datasets were generated or analysed during the current study.
